# Effects of Stress on Phase Transformations in Grinding by FE Modeling and Experimental Approaches

**DOI:** 10.3390/ma12142327

**Published:** 2019-07-22

**Authors:** Shichao Xiu, Yansheng Deng, Xiangna Kong

**Affiliations:** Mechanical Engineering & Automation, Northeastern University, Shenyang 110819, China

**Keywords:** grinding, phase transformation, strengthened layer, stress-induced phase transformation, thermal–mechanical–metallurgical coupling, material processing, phase evolution

## Abstract

In the grinding process, the materials within the surface layer may undergo phase transformation and finally form a strengthened layer. It is of great significance to model the phase transformation and predict the characteristics of the strengthened layer accurately. The phase transformations occur under the varying temperature and high stress–strain in grinding, so the effects of stress on the transformations are inescapable. This paper focuses on revealing the effects of stress on phase transformations in grinding. For this purpose, a thermal–mechanical–metallurgical direct coupling finite element (FE) model of grinding was established in Abaqus. The coupling interactions such as the latent heat, the volume change strain caused by phase transformation, and the stress-induced phase transformation were considered in the modeling procedure. Grinding experiments were carried out and proved the model could accurately predict the microstructure distribution and thickness of the strengthened layer. Further, the evolution of the phase transformation was discussed, and the effects of stress on the transformations were revealed.

## 1. Introduction

Grinding is a widely used machining process in engineering because it can create a precise surface and a surface layer with good surface integrity [1]. In the process, there is a large amount of heat transfer into the workpiece, so the temperature of the surface layer rises sharply and a great temperature gradient forms within the layer. The grinding heat is the main reason for the phase transformation and the formation of the strengthened layer. The grinding heat is usually considered as a negative factor, and various cooling techniques are used to cool the workpiece. In 1994, German scholars proposed a new technology called grinding hardening, in which the grinding heat is directly used to quench the surface material [2]. In fact, whether in traditional grinding or grinding hardening, surface materials may undergo phase transformation because of the excessive temperature, so studying the phase transformation in grinding has universal significance. 

Some theoretical and experimental studies about phase transformation and strengthened layer in grinding have been carried out. Brinksmeier and Brokhoff [2] first found the microstructure of the surface layer after grinding could be divided into two layers. The nearest surface is a white etching layer a few microns thick, and the next layer is the strengthened layer comprised of martensite and carbides. Furthermore, they pointed out the materials undergo short austenitization and self-quenching. Zarudi and Zhang [3,4] took the quenched steel as the experimental material and carried out grinding hardening experiments. They observed the strengthened layer by a transmission electron microscope (TEM) and found there was no ferrite in the depth range of 0–0.9 mm, but when the depth was greater than 0.3 mm, bainite appeared. Then, they thought the microstructure composition of the strengthened layer was complex, the nearest surface layer was the refined martensite, and more and more bainite appeared with increasing depth. Nguyen and Zhang found the martensite was finer after cooling with liquid nitrogen in grinding hardening by observing the microstructure of the surface layer. This is because the temperature is lower than the room temperature after the usage of the liquid nitrogen, and the martensite transformation occurs below the room temperature [5]. Mao et al. [6] studied the formation of the white layer in grinding AISI 52100 steel. Furthermore, they found the phase transformation, retained austenite, and white layer could occur below the nominal temperature of the phase transformation. They thought the plastic deformation caused by the mechanical effect could affect the formation of the white layer. Liu et al. [7] studied the effects of grinding depth and initial microstructure of the workpiece on the depth, microstructure, and micro-hardness distribution of the strengthened layer in grinding AISI 1060 steel. The results show the strengthened layer can divide into the complete hardening region and the transition region. The complete hardening region consists of martensite, retained austenite, and little cementite. Furthermore, the grinding depth and initial microstructure have no obvious effect on the martensite morphology and micro-hardness in the complete hardening region. However, the depth of the strengthened layer increases with the grinding depth or uniformity of the initial microstructure increase.

Simulation is economical, efficient, and beneficial to understanding the process mechanism. The simulation of phase transformation is widely used in heat treatment, welding, and other research [8,9]. Since the phase transformation mainly depends on temperature, it is necessary to simulate the temperature. Based on finite element (FE) thermal analysis, Zhang and Mahdi [10] studied the effects of feeding speed, heat flux, material thermal properties, and convective ability of cooling medium on phase transformation in grinding. Brosse et al. [11] used the FE software Sysweld in which the Leblond’s transformation model is adopted to calculate the phase transformation in grinding. Ding et al. [12] theoretically analyzed the phase transformation of martensitic steel in grinding. Moreover, they considered that temperature is not the only factor affecting the phase transformation in grinding, and the strain rate also plays an important role. Therefore, they established a phase transformation model incorporating the effect of strain rate based on the experimental data and regression analysis. Deng et al. proposed a microstructure evolution model based on cellular automata (CA), and then in detail discussed the austenite transformation in grinding [13]. Salonitis [14] first proposed a hybrid CA–FE model for simulating the phase transformation in grinding hardening, then discussed the grain size and the phase composition. 

So far, most of the studies of the phase transformation in grinding are experimental and theoretical. The existing models and simulations also need to improve due to: (1) the temperature, stress–strain, and phase transformation are directly coupled in grinding, but most studies ignore or simplify the coupling relations; and (2) the surface materials undergo austenite transformation during the heating period, and may undergo various phase transformations during the cooling period, but these transformations are not always fully accounted for. This paper focuses on revealing the effects of stress on the phase transformations in grinding. The following works were carried out: first, the thermal and mechanical loads, initial and boundary, in the grinding were analyzed or modeled. The phase transformation incorporating the effects of stress in grinding was analyzed theoretically and modeled mathematically; then, the thermal–mechanical–metallurgical model was implemented by developing several user subroutines in Abaqus. Next, the reliability of the model was verified by conducting grinding experiments of AISI 1045 steel. The microstructure distribution and the thickness of the strengthened layer were observed or measured. Finally, the evolutions of the temperature and the phase transformation in grinding are discussed, and the effects of stress on phase transformations are revealed.

## 2. Thermal–Mechanical–Metallurgical Coupling Model of Grinding

### 2.1. Thermal–Mechanical Model of Grinding 

In grinding, the grinding force and heat are the causes of deformation, material removal, and phase transformation. The heat conduction satisfies the following differential equation:

(1) $$\rho C_{p}\frac{\partial T}{\partial t}-\frac{\partial }{\partial x}\left(k\frac{\partial T}{\partial x}\right)-\frac{\partial }{\partial y}\left(k\frac{\partial T}{\partial y}\right)-\frac{\partial }{\partial z}\left(k\frac{\partial T}{\partial z}\right)-\dot{Q}=0$$

where ρ, $C_{p}$, and k are the density, the specific heat, and the heat conductivity of the workpiece, respectively. Table 1 shows the temperature-dependent ρ, $C_{p}$, and k used in the present work. $\dot{Q}$ is the heat flux of the inner heat source. Considering the release or absorption of heat due to phase transformation, $\dot{Q}$ can be estimated by [15]:

(2) $$\dot{Q}=\sum H_{x}\frac{df_{x}}{dt}$$

where $H_{x}$ is the changing latent heat per unit transformed volume, and $df_{x}$ is the increment of transformed volume during the time increment dt. Subscript x in $H_{x}$ and $df_{x}$ refers to the transformation type. Table 2 shows $H_{x}$ used in the present work [16]. The subroutine *HETVAL provided in Abaqus is used and written to calculate the latent heat of phase transformation.

To solve Equation (1), it is necessary to define the initial and boundary conditions in grinding. The initial temperature of the workpiece is equal to the room temperature:

(3) $$T\left(x,y,z\right)|{}_{t=0}=T_{\infty }=20{}^{\circ}C$$

**Figure 1:**
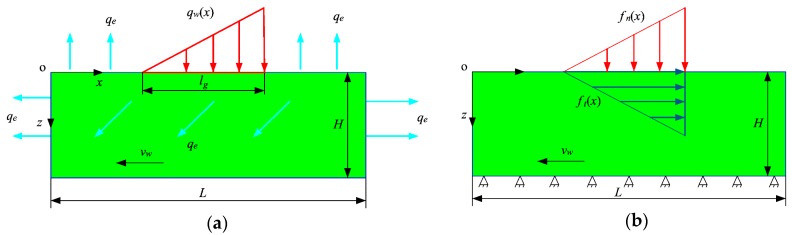
Boundary conditions in the grinding: (**a**) thermal boundary conditions; (**b**) mechanical boundary conditions. H is the height of the workpiece, L is the length of the workpiece, $q_{e}$ is the equivalent heat flux of the convection and radiation.

Figure 1a shows the diagram of the thermal boundary conditions in grinding. The distributed heat source moves and acts on the workpiece surface, and it is assumed that the heat source is distributed in a right triangle:

(4) $$q_{w}\left(x\right)=2q_{m}\frac{\left(x-v_{w}t\right)+l_{g}}{l_{g}},v_{w}t-l_{g}\leq x\leq v_{w}t$$

where $q_{w}$ is the heat flux of the heat source, $v_{w}$ is the feeding speed and $l_{g}$ is the contact chord length, which can be estimated by $\sqrt{a_{p}d_{s}}$, where $d_{s}$ is the wheel diameter and $a_{p}$ is the grinding depth. $q_{m}$ is the mean heat flux within the contact zone and can be expressed as:

(5) $$q_{m}=\varepsilon \frac{F_{t}v_{s}}{bl_{g}}$$

where $F_{t}$ is the tangential grinding force, $v_{s}$ is the wheel speed, and b is the grinding width. ε is the heat partition ratio transferred into the workpiece and can be estimated by [17]:

(6) $$\varepsilon =\frac{1}{{\left[1+\frac{{\left(k\rho c\right)}_{s}v_{s}}{{\left(k\rho c\right)}_{w}v_{w}}\left(\frac{A_{R}}{A_{s}}\right)\right]}^{1/2}}$$

where ${\left(k\rho c\right)}_{s}$ and ${\left(k\rho c\right)}_{w}$ are the thermal properties of the wheel and the workpiece, respectively. $A_{R}$ and $A_{s}$ are real and geometrical contact areas between the workpiece and the wheel, respectively. Furthermore, the heat convection and heat radiation occur on the workpiece surfaces, and the equivalent heat flux of the convection and radiation is:

(7) $$q_{e}=h_{c}\left(T_{s}-T_{\infty }\right)+\sigma_{b}\varepsilon_{b}\left(T_{s}{}^{4}-T_{\infty }{}^{4}\right)$$

where $h_{c}$ is the convection coefficient, $T_{s}$ is the surface temperature, $\varepsilon_{b}$ is the emissivity (0.21), and $\varepsilon_{b}$ is the Stefan–Boltzmann constant (5.67 × 10^−8^ W/(m^2^ °C^4^)).

In grinding, the surface materials have elastic–plastic deformation due to the actions of the grinding force and heat. According to Prandtl–Reuss plastic increment theory, the constitutive relationship within the elastic zone is:

(8) $$d\left\{\sigma \right\}={\left[D\right]}_{e}d\left\{\varepsilon \right\}-{\left[C\right]}_{e}dT$$

and in the plastic zone is:

(9) $$d\left\{\sigma \right\}={\left[D\right]}_{ep}d\left\{\varepsilon \right\}-{\left[C\right]}_{ep}dT$$

where ${\left[D\right]}_{e}$ and ${\left[D\right]}_{ep}$ are the elastic matrix and plastic matrix, respectively, and ${\left[C\right]}_{e}$ and ${\left[C\right]}_{ep}$ are, respectively, elastic vector and plastic vector related to the temperature, and dT is the temperature increment. d{σ} and d{ε} are the total stress increment and strain increment, respectively. The major strain sources, such as mechanical strain, thermal strain, and the volume change strain caused by phase transformation, were considered, and some less important strain sources, such as stress relaxation and strain caused by transformation plasticity, were ignored in the present paper [18,19]. Then, d{ε} can be expressed as:

(10) $$d\left\{\varepsilon \right\}=d\left\{\varepsilon^{e}\right\}+d\left\{\varepsilon^{p}\right\}+d\left\{\varepsilon^{th}\right\}+d\left\{\varepsilon^{\Delta V}\right\}$$

where $d\left\{\varepsilon^{e}\right\}$ is the elastic strain, which can be calculated by Hooke’s law with the temperature-dependent elastic modulus E and constant Poisson’s ratio v (as shown in Table 3). $d\left\{\varepsilon^{p}\right\}$ is the plastic strain increment following Prandtl–Reuss flow rule, the von Mises yield criterion, and the Johnson-Cook model. Table 4 shows the Johnson-Cook coefficients used in the present work [20]. $d\left\{\varepsilon^{th}\right\}$ is the thermal strain increment and can be estimated by:

(11) $$d\left\{\varepsilon^{th}\right\}=\alpha dT$$

where α is the thermal expansion coefficient; the temperature-dependent α used in the present work is listed in Table 3. $d\left\{\varepsilon^{\Delta V}\right\}$ is the volume strain increment caused by phase transformation and can be estimated by [21]:

(12) $$d\left\{\varepsilon^{\Delta V}\right\}=\frac{1}{3}\frac{\Delta V}{V}df_{x}$$

where ΔV/V is the volume change ratio caused by phase transformation and is listed in Table 5 [21]. $d\left\{\varepsilon^{\Delta V}\right\}$ was incorporated into the model by using and writing the subroutine *UEXPAN. 

Figure 1b shows the mechanical boundary conditions in grinding. The upper surface is under the actions of moving normal grinding force and tangential grinding force. The grinding forces distribute in the contact zone in a right triangle and can be expressed as follows:

(13) $$f_{t}\left(x\right)=2\frac{F_{t}}{bl_{g}}\frac{\left(x-v_{w}t\right)+l_{g}}{l_{g}},v_{w}t-l_{g}\leq x\leq v_{w}t$$

(14) $$f_{n}\left(x\right)=2\frac{F_{n}}{bl_{g}}\frac{\left(x-v_{w}t\right)+l_{g}}{l_{g}},v_{w}t-l_{g}\leq x\leq v_{w}t$$

where $F_{n}$ is the normal grinding force. The bottom surface is fixed on the worktable, and its displacements are set as 0.

### 2.2. Metallurgical Model for Grinding 

#### 2.2.1. Diffusive Transformations

**Figure 2:**
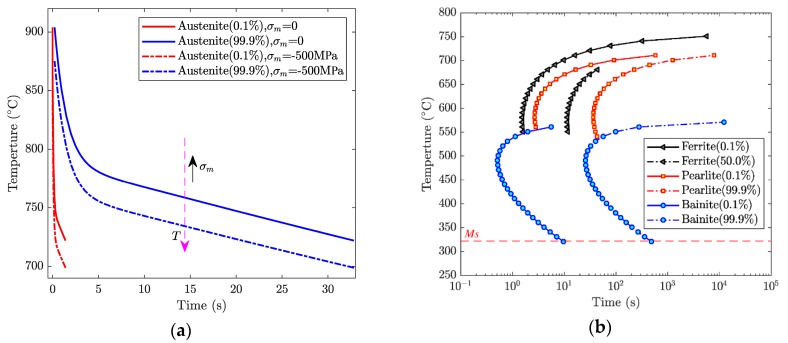
Transformation kinetic curves of AISI 1045 steel: (**a**) time–temperature-austenitization (TTA) diagram; (**b**) time–temperature-transformation (TTT) diagram.

For the diffusive transformations, such as austenite transformation, ferrite transformation, pearlite transformation, and bainite transformation under isothermal condition, a kinetic equation proposed by Avrami is widely used [15,22]:

(15) $$f_{x}=1-\exp\left(-b{\left(t-\tau \right)}^{n}\right)$$

where $f_{x}$ is the transformation fraction, t is isothermal time, τ is the incubation time, b is the growth factor, and n is the growth index. b and n at a certain isothermal temperature can be estimated by:

(16) $$n=\frac{\ln\left[\frac{\ln\left(1-f_{x1}\right)}{\ln\left(1-f_{x2}\right)}\right]}{\ln\left(\frac{t_{1}}{t_{2}}\right)}$$

(17) $$b=-\frac{\ln\left(1-f_{x1}\right)}{t_{1}^{n}}$$

where $f_{x1}$ and $f_{x2}$ are two different transformation fractions at an isothermal temperature, and their corresponding isothermal time are $t_{1}$ and $t_{2}$. $f_{x1}$ and $f_{x2}$ are usually set, respectively, as 0.1% and 99.9% in calculations. $f_{x1}$, $f_{x2}$, $t_{1}$, and $t_{2}$ can be obtained from a time–temperature-austenitization (TTA) diagram (isothermal kinetic curves of austenite formation) or time–temperature-transformation (TTT) diagram (isothermal transformation curves of undercooled austenite). Figure 2 shows the TTA and TTT diagrams of AISI 1045 steel used in the present paper. The TTT diagram was directly obtained from JmatPro and the TTA diagram was transformed from the CHA diagram (continuous heating kinetic curves of austenite formation), which was also obtained from JmatPro (Public Release Version 7.0.0, Sente Software Ltd., Guildford, United Kingdom). 

According to Cheng’s work [23], the temperature-dependent b and n can be obtained by fitting in the following two forms:

(18) $$\ln b\left(T\right)=C_{3x}T^{3}+C_{2x}T^{2}+C_{1x}T+C_{0x}$$

(19) $$n\left(T\right)=D_{3x}T^{3}+D_{2x}T^{2}+D_{1x}T+D_{0x}$$

where $C_{0x,1x,2x,3x}$ and $D_{0x,1x,2x,3x}$ are constants. The incubation time τ related to temperature can also be obtained by fitting in the form:

(20) $$\ln\tau \left(T\right)=E_{3x}T^{3}+E_{2x}T^{2}+E_{1x}T+E_{0x}$$

where $E_{0x,1x,2x,3x}$ are constants.

To describe the phase transformation under non-isothermal condition, Scheil’s additivity rule based on fictitious time is used [24,25]. A non-isothermal process could be divided into many isothermal steps with a time interval Δt; the transformed fraction after the ith. isothermal step is:

(21) $$f_{i}=1-\exp\left(-b{\left(t_{i}^{*}+\Delta t\right)}^{n}\right)$$

where $t_{i}^{*}$ is the fictitious time used in the ith isothermal step:

(22) $$t_{i}^{*}={\left(-\frac{\ln\left(1-f_{i-1}\right)}{b}\right)}^{1/n}$$

where $f_{i-1}$ is the transformed fraction after the i−1th. isothermal step.

#### 2.2.2. Martensite Transformation

Martensite transformation occurs when the temperature is less than the starting temperature of martensite transformation Ms in the cooing period. The undercooled austenite constantly transforms into martensite with the temperature decreasing. The transformed fraction can be calculated by the Koistinen–Marburger equation [26]:

(23) $$f_{M}=f_{\gamma }^{*}\left(1-\exp\left(-\alpha \left(Ms-T\right)\right)\right)$$

where $f_{M}$ is the formed martensite fraction, $f_{\gamma }^{*}$ is the retained austenite fraction before martensite transformation, and α is a constant depending on the material and is equal to 0.011 for most Fe–C alloy steels.

#### 2.2.3. Effect of Stress on Phase Transformation

Austenite transformation and martensite transformation are dominant in grinding, so the effects of stress on the two transformations were considered in the present work. For AISI 1045 steel, if ignoring the effect of stress, the austenite transformation begins when the temperature exceeds the nominal equilibrium temperature A1(722.0 °C). However, the transformation in grinding happens under high stress, which could change A1. The Clausius–Clapeyron equation can be used to incorporate the effect of stress [27,28]. Material is under a complex stress state, so the pressure in the Clausius–Clapeyron equation is replaced by the hydrostatic stress:

(24) $$\frac{d\left(-\sigma_{m}\right)}{dT}=\frac{\Delta H_{\alpha \gamma }}{T\Delta V_{\alpha \gamma }}$$

where $\sigma_{m}$ is the hydrostatic stress, and $\sigma_{m}=\left(\sigma_{1}+\sigma_{2}+\sigma_{3}\right)/3$ and $\sigma_{m}<0$ indicate that the stress is compressive. $\Delta H_{\alpha \gamma }$ (920.5J/mol) is the enthalpy of ferrite–austenite transformation per mole, and $\Delta V_{\alpha \gamma }$ ($-0.06{\mathrm{cm}}^{3}/\mathrm{mol}$) [28] is the volume change of the transformation per mole:

(25) $$\Delta V_{\alpha \gamma }=\frac{M}{\rho_{\gamma }}-\frac{M}{\rho_{\alpha }}$$

where $\rho_{\alpha }$ and $\rho_{\gamma }$ are the densities of ferrite and austenite, respectively. Through deduction and integration of Equation (25), the equilibrium temperature A1 incorporating the effect of stress expresses as:

(26) $$T=T_{0}\exp\left(\frac{\Delta V_{\alpha \gamma }\left(-\sigma_{m}\right)}{\Delta H_{\alpha \gamma }}\right).$$

Besides A1, the stress also changes the equilibrium temperatures A3. For example, Figure 2a shows A1 and A3 under the action of $\sigma_{m}=-500\mathrm{Mpa}$ (minus represents compressive stress). From Figure 2b, the kinetic curves have movement in the temperature decreasing direction, which indicates the hydrostatic compressive stress could promote the austenite transformation by reducing A1 and A3.

The original TTT and TTA diagrams obtained from JMatPro are used to calculate transformation coefficients, and then the transformation without the effect of stress can be calculated by Equations (21) and (22) with the coefficients. In order to incorporate the effect of stress on the transformation, the TTT and TTA diagrams are adjusted according to Equations (24)–(26), then the adjusted TTT and TTA diagrams are used to calculate transformation coefficients, and finally, the transformation with the effect of stress is calculated by Equations (21) and (22) with the coefficients.

When the mechanical driving force produced by the internal stress and chemical driving force exceeds a critical value, martensite transformation will occur beyond the nominal starting temperature Ms (321.9 °C). This is called stress-induced martensite transformation [29]. In general, the effects of stress on martensite transformation include the effect of hydrostatic stress and the effect of uniaxial stress. The hydrostatic compressive stress decreases the equilibrium temperature Ms, and both uniaxial tension stress and compression stress increase the equilibrium temperature Ms. To calculate the effects of shear stress and normal stress on Ms under complex stress conditions, Inoue proposed a model for calculating the variation of Ms [30,31]:

(27) $$\Delta M_{S}=A\sigma_{m}+B\sqrt{J_{2}}$$

where $J_{2}$ is second deviatoric invariant of the stress tensor and can be expressed as:

(28) $$J_{2}=\left(1/6\right)\left({\left(\sigma_{1}-\sigma_{2}\right)}^{2}+{\left(\sigma_{2}-\sigma_{3}\right)}^{2}+{\left(\sigma_{3}-\sigma_{1}\right)}^{2}\right)$$

A (0.05) and B (0.033) are coefficients related to the material [28].

### 2.3. Model Implementation in FE

The workpiece was modeled with the size of 50 mm (length) × 16 mm (height). Since the phase transformation only occurs within the shallow surface layer in the grinding, the depth range of 0–1 mm of the workpiece was finely meshed and the rest was coarsely meshed. The bottom of the workpiece was fixed on workbench. The heat convection and heat radiation were applied on the surfaces by writing subroutine *FILM and setting emissivity. User subroutines *DFLUX, *DLOAD, and *UTRACLOAD were defined to achieve the applications of moving grinding heat flux, normal grinding force, and tangential grinding force, respectively. The purpose of this paper was to establish a thermal–mechanical–metallurgical direct coupling FE model for grinding and reveal the effects of stress on the phase transformation. However, the Abaqus does not provide the model of thermal–mechanical–metallurgical coupling analysis. In order to accomplish the coupling analysis, the temp-displacement coupling analysis was chosen and several user subroutines were used. The phase transformations were calculated by defining user subroutine *USDFLD, the latent heat of the phase transformation was defined by writing user subroutine *HETVAL, and the volume change strain caused by phase transformation was defined in user subroutine *UEXPAN.

## 3. Experimental Details

**Figure 3:**
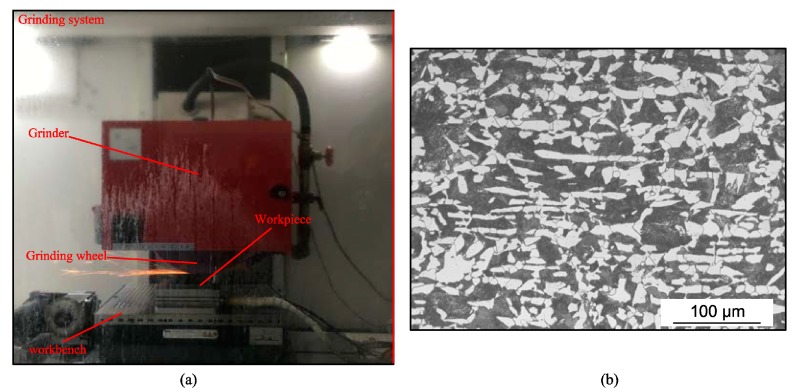
(**a**) Experimental setup; (**b**) initial microstructure of AISI 1045 steel.

The grinding experiments were conducted to validate the model. Figure 3a shows the experiment setup. The grinder used was the BLOHM ORBIT 36 plane and forming grinder (ORBIT 36, United Grinding (Shanghai) Ltd., Shanghai, China). 

The material used in the experiments was AISI 1045 steel (Anshan Steel Group, Anshan, China), the chemical composition of which is shown in Table 6. Figure 3b shows the initial microstructure of the material, and the material consisted of 56.3% pearlite and 43.7% ferrite. The dimensions of the grinding region of the specimen were 50 mm (length) × 10 mm (width) × 20 mm (height). An aluminum oxide wheel with a grain size number of 60, diameter of 340 mm, and width of 40 mm, was used.

Table 7 shows the experimental grinding parameters used in present work.

To obtain a metallographic, a part of each ground workpiece was first cut as the metallographic sample. Then, the samples had treatments with lapping, polishing, cleaning, and corrosion. Finally, the microstructure distribution and thickness of the strengthened layer were observed or measured by the LEICA-DMIRM multifunctional metallographic microscope (LEICA-DMIRM, Leica Microsystems Inc., Buffalo Grove, United States).

## 4. Results and Discussion

### 4.1. Comparison of Experimental Results and Simulated Results

**Figure 4:**
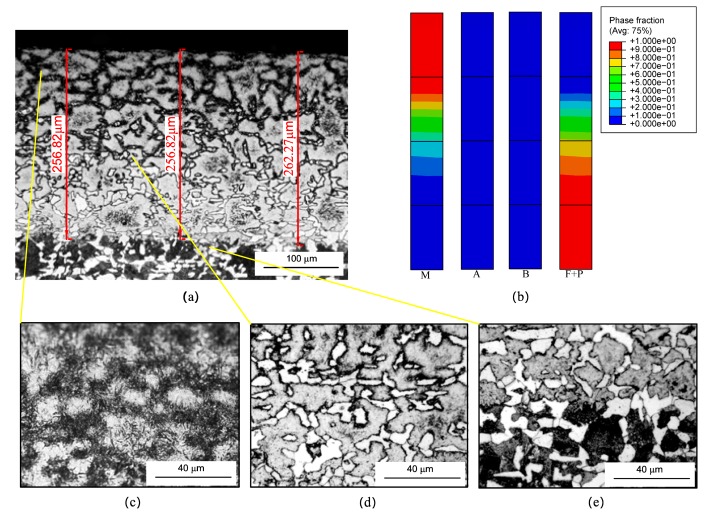
Microstructure distribution of the strengthened layer under no. 1 grinding parameter: (**a**) experimental result; (**b**) simulated result; (**c**) the complete strengthened layer; (**d**) the transition layer; (**e**) the boundary between the strengthened layer and the bulk region.

Figure 4 shows the microstructure distribution of the strengthened layer under the no. 1 grinding parameter. Figure 4a is a metallographic obtained by experimental observation and Figure 4b is a map obtained from the simulation. Considering the phase transition occurs only in the shallow surface layer, only four elements nearest to the surface are selected to show the simulated results. As seen in Figure 4a, the microstructure of the strengthened layer was obviously different from the original microstructure. The ferrite was bright white, martensite was bright black, and the pearlite was black under current corrosion conditions. The strengthened layer visually presented dark–bright–dark with the depth increasing because of the different degrees of phase transformation along the depth direction. The strengthened layer could be divided into complete strengthened layer and transition layer according to the degree of phase transformation. The upper dark layer almost comprised of 100% martensite was the complete strengthened layer (Figure 4c), and the next bright layer comprised of martensite and pearlite + ferrite was the transition layer (Figure 4d). In the deeper depth, the materials did not undergo phase transformation, and the boundary between the strengthened layer and the bulk region is shown in Figure 4e. As seen in Figure 4a, the martensite decreased and the ferrite + pearlite increased along the depth within the strengthened layer. As seen in Figure 4b, the retained austenite and bainite of the strengthened layer were small and changeless along the depth, so we only discuss the variations of the martensite and ferrite + pearlite. Furthermore, the martensite fraction was about 90–100% within the depth range of 0–120 μm. This shows the materials within the range underwent complete austenite transformation during the heating period and mainly underwent martensite transformation during the cooling period. Comparing to the experimental result (Figure 4a), the range corresponded to the complete strengthened layer. There were martensite and ferrite + pearlite within the depth range of 120–250 μm, and the martensite increased and ferrite + pearlite decreased with the increases in depth. The range corresponded to the transition layer in Figure 4a. In summary, the simulated microstructure distribution was consistent with the experimental result.

**Figure 5:**
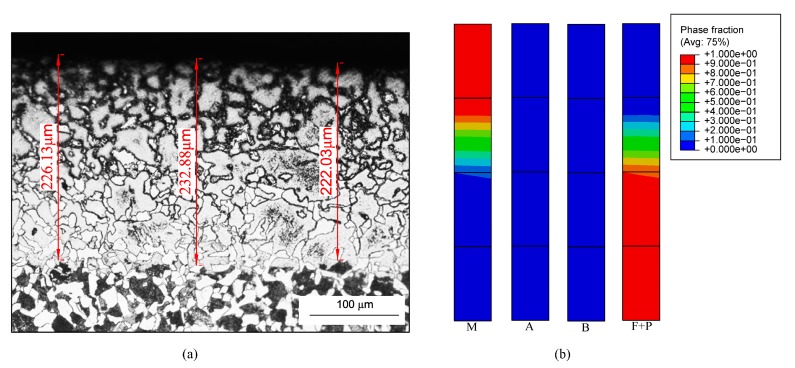
Microstructure distribution of the strengthened layer under no. 2 grinding parameter: (**a**) experimental result; (**b**) simulated result.

**Figure 6:**
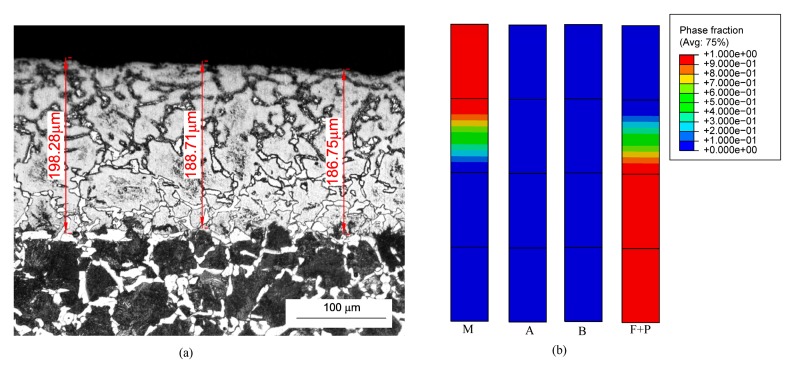
Microstructure of the strengthened layer under no. 3 grinding parameter: (**a**) experimental result; (**b**) simulated result.

**Figure 7:**
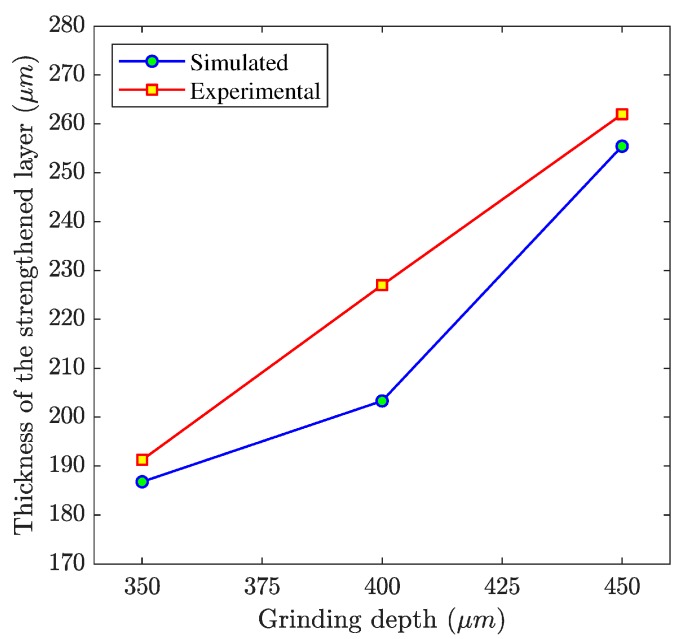
The thickness of the strengthened layer under different grinding depths.

Figure 5 and Figure 6 show the microstructure distribution of the strengthened layer under the no. 2 and no. 3 grinding parameters. These shows the microstructure distributions under the two grinding parameters were similar to that under the no. 1 grinding parameter. However, the thicknesses of the strengthened layers were different under different grinding parameters. The predicting accuracy of the established model could be further verified by comparing the thicknesses of the strengthened layers. In the experiments, to overcome the measuring error and the variability of the thickness, we measured the thickness at three different positions by a microscope, and used the average to compare with the simulated results. Figure 7 shows the comparison results of the thicknesses. This means that the simulated thicknesses were in good agreement with the experimental results and the maximum error was 10.6%.

### 4.2. Temperature and Phase Distributions

**Figure 8:**
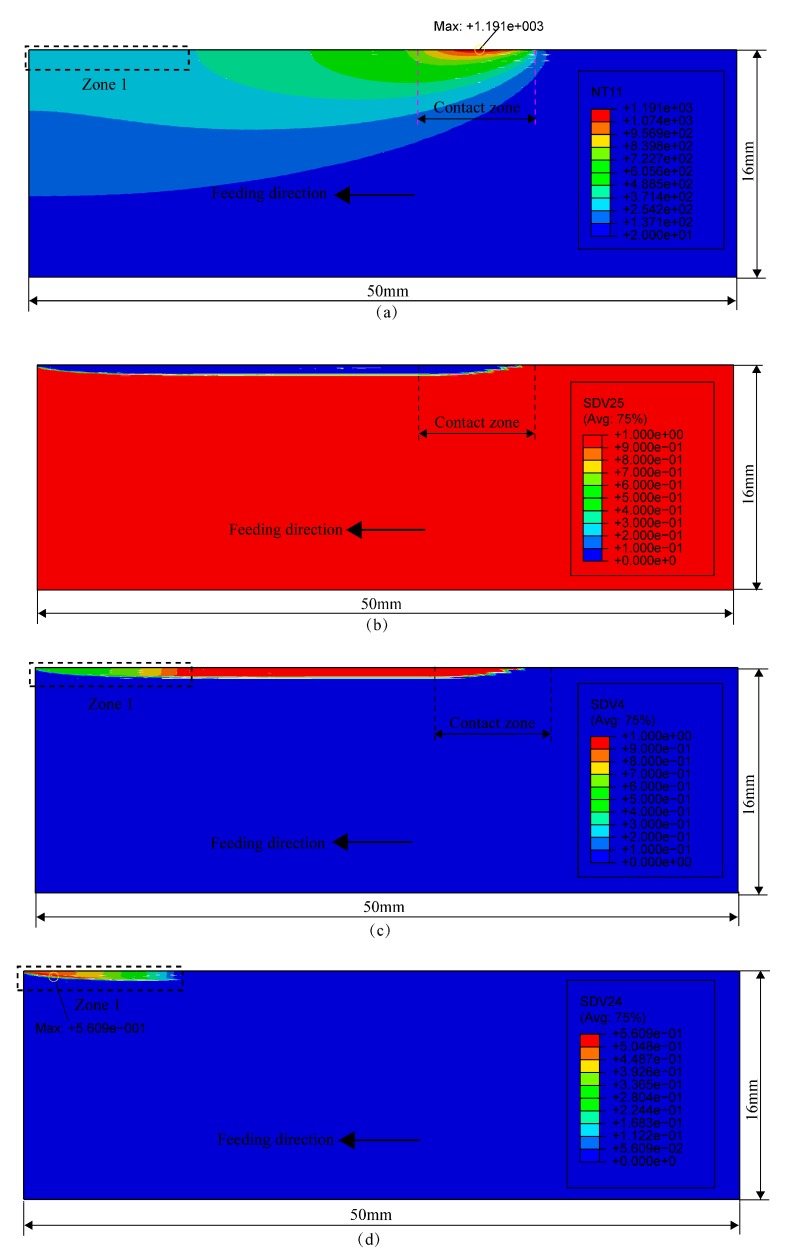
Temperature and microstructure distributions at the moment of 3.576 s: (**a**) temperature; (**b**) ferrite + pearlite; (**c**) austenite; (**d**) martensite.

Figure 8a shows the temperature distribution at the moment of 3.576 s under the grinding parameters, $a_{p}=200\mu m$, $v_{w}=0.6m/\min$, and $v_{s}=30m/s$. It shows a temperature field with a large gradient formed in the surface layer of the workpiece. The maximum temperature appeared on the workpiece surface in the contact zone, reaching 1191 °C. In the rear region of the contact zone, the temperature was generally higher, which was due to the heat source having just passed. In the region farther away from the contact zone, the grinding heat source had passed for some time, so the temperature was lower. In the front region of the contact zone, the temperature was only high around the contact zone, but close to room temperature at a farther position because the heat source had not passed and the heat could not transfer to a deeper and farther position.

Figure 8b–d show the distributions of ferrite + pearlite, austenite, and martensite at the moment of 3.576 s. As seen in Figure 8b,c, the fractions of ferrite + pearlite and austenite of the region in and behind the contact zone were 0–10% and 90–100%, respectively. This means that the materials in the region underwent austenite transformation. In particular, as shown in Figure 8d, martensite appeared in zone 1 with a maximum fraction of 56.1%. This shows that the martensite transformation began in zone 1. This was because the temperature of the materials in the zone dropped below 300 °C at this moment, as shown in Figure 8a.

### 4.3. Evolution of the Phase Transformation

**Figure 9:**
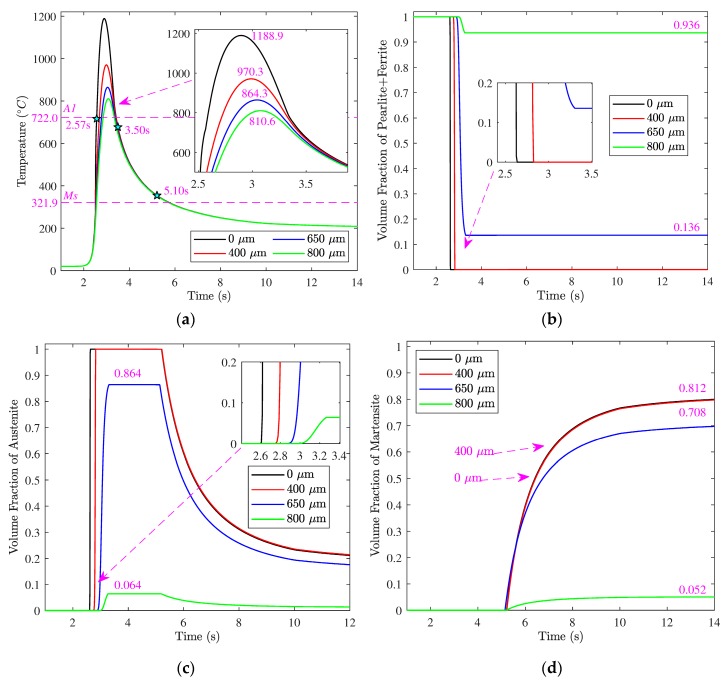
Evolution of temperature and each phase at different depths: (**a**) temperature; (**b**) ferrite + pearlite; (**c**) austenite; (**d**) martensite.

Figure 9a shows the time–temperature histories of four material points at different depths. The maximum temperatures were 1188.9, 970.3, 864.3, and 810.6 °C, respectively, which exceeded the actual starting temperature A1. The temperature increased rapidly during the temperature rising period and the temperature dropping rate was also high during the cooling period. The type of phase transformation occurring during the cooling period was relevant to the temperature dropping rate, so the average rates within 400–800 °C were calculated and used to judge the possible phase transformation. The average rates at different depths were within 260–300 °C/s, which were higher than the critical temperature dropping rates of bainite transformation of AISI 1045 steel, which is 100 °C/s. This illustrates the undercooled austenite would not experience ferrite transformation, pearlite transformation, and bainite transformation before martensite transformation. 

Figure 9b–d show the evolution of each phase at different depths. We ignored the evolution of the bainite because the simulation results showed that bainite transformation rarely occurs. The austenite transformation started since the temperature reached the actual A1. For the material consisting of pearlite and ferrite grains, the pearlite first underwent austenite transformation, and the ferrite underwent austenite transformation after pearlite dissolution. Figure 9c shows the austenite fractions increased over time within a short time range after the austenite transformation began. Closer to the surface, the austenite transformation began earlier, which was due to the temperature of the material closer to the surface rising to A1 earlier. Austenite transformation is a time-consuming process. Complete austenite transformation occurs only when the temperature lasts long enough above A1. At the depths of 0 μm and 400 μm, complete austenite transformation happened and forms 100% austenite at the moments of 2.636 s and 2.836 s, respectively. At the depth of 650 μm, the austenite transformation was incomplete, and formed 86.4% austenite. Furthermore, as seen in Figure 9b, all pearlite transformed into austenite while the remaining 13.6% ferrite did not transform into austenite at the depth. The austenite transformation was also incomplete, and only formed 6.4% austenite at the depth of 800 μm. As seen in Figure 9b, the remaining 93.6% pearlite + ferrite did not transform into austenite at the depth. This is because the duration times beyond A1 at the depths of 650 and 800 μm were not enough. 

As seen in Figure 9c, the fractions of the austenite at all the depths were stable for a period during which the factions of other phases were changeless. This indicates no phase transformation took place during the period. When the temperature dropped below Ms, the martensite transformation began. As seen in Figure 9d, the martensite transformations took place almost at the same time at different depths. At the depths of 0 and 400 μm, the evolution curves of martensite almost coincided, because their temperatures below Ms almost coincided during the cooling process, as shown in Figure 9a. The fraction of austenite decreased and the fraction of martensite increased over time because of the continual decline of the temperature. At the moment of 14 s, the fractions of martensite at the depths were about 81.2%, 81.2%, 70.8%, and 5.2%, respectively.

### 4.4. Effect of Stress on Phase Transformation in Grinding

**Figure 10:**
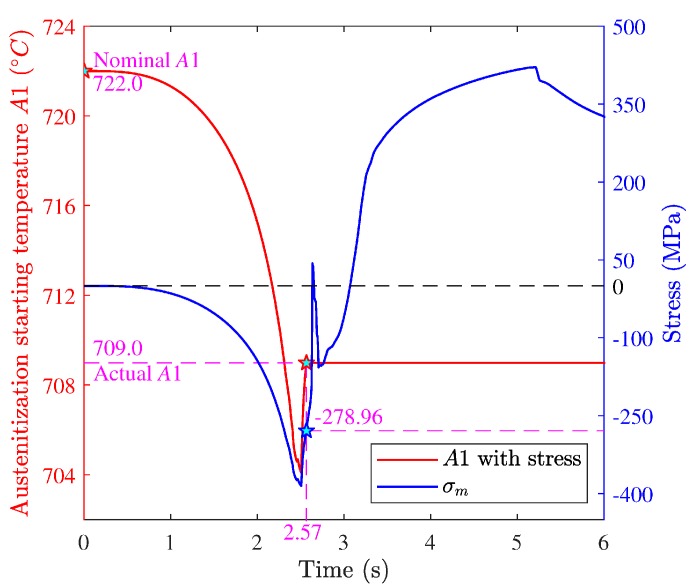
Hydrostatic stress histories and A1 with stress.

Figure 10 shows the hydrostatic stress history at the depth of 0 μm during grinding and A1 considering the stress. In the figure, negative stress represents compressive stress and positive stress represents tensile stress. It shows the hydrostatic stress was compressive within the time range of 0–2.57 s (a period before austenite transformation), and firstly increased and then decreased in value over time. As such, A1 with stress decreased relative to the nominal A1, and the changing trend was consistent with the hydrostatic stress. This is because hydrostatic compressive stress reduces A1. Figure 9a shows the temperature of the material point kept rising within the time range of 0–2.57 s and exceeded the equilibrium temperature A1 at the moment of 2.57 s, and then the austenite transformation began. The hydrostatic stress was −278.96 MPa at the moment, and A1 with stress was 709.0 °C (the actual starting temperature of austenite transformation for the material point). Compared to the nominal A1, the actual A1 decreased by 13.0 °C due to the effect of the hydrostatic compressive stress.

**Figure 11:**
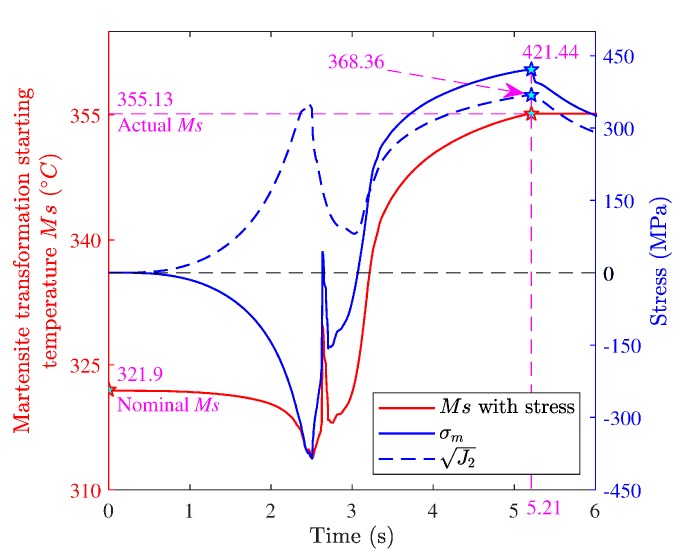
Hydrostatic stress and $\sqrt{J_{2}}$ histories, and Ms with stress.

Figure 11 shows the hydrostatic stress history and $\sqrt{J_{2}}$ history of the material point at the depth of 0 μm during grinding and the martensite transformation starting temperature Ms with stress. As seen in the figure, the hydrostatic stress was tensile in the time range of 3.50–5.21 s (a period before martensite transformation), and increased over time. $\sqrt{J_{2}}$ also increased over time in the time range. Ms with stress increased relative to the nominal Ms in the time range due to the effects of hydrostatic tensile stress and $\sqrt{J_{2}}$, and increased over time. This is because both hydrostatic tension stress and $\sqrt{J_{2}}$ could increase Ms. As seen in Figure 9a, the temperature of the material point decreased during the period. The temperature began to be lower than the equilibrium temperature Ms at the moment of 5.21 s, and the martensite transformation began. At the moment, the hydrostatic stress was 421.44 MPa, $\sqrt{J_{2}}$ was 368.36 MPa, and Ms with stress was 355.13 °C (actual starting temperature of martensite transformation of the material point). Compared with the nominal Ms, the actual Ms increased by 33.23 °C due to the effects of hydrostatic tensile stress and $\sqrt{J_{2}}$.

**Figure 12:**
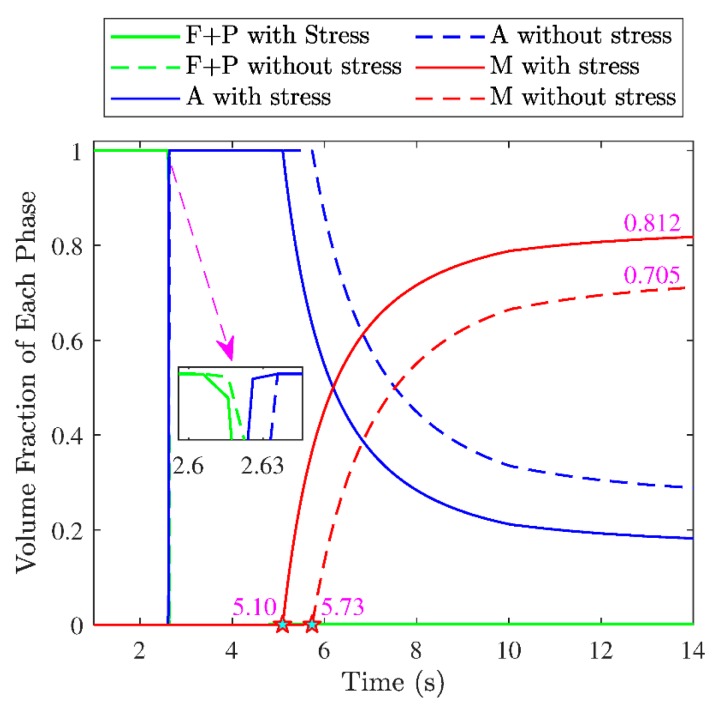
Effect of stress on the evolution of each phase.

In order to analyze the effects of stress on the phase transformations and the final characteristics of the strengthened layer in the grinding, the simulations were carried out with and without considering the effect of stress. Figure 12 shows the evolution of each phase in grinding with and without considering stress. As seen in the figure, no matter whether considering the stress or not, the material point underwent complete austenite transformation and resulted in 100% austenite. The difference was the austenite transformation began earlier when considering stress. This is because hydrostatic stress is compressive and reduces A1. Then, the austenite transformation occurred at lower temperature. The martensite transformation took place earlier with considering the stress than without considering the stress. This was because both the hydrostatic tensile stress and $\sqrt{J_{2}}$ could increase Ms; then the martensite transformation occurred at a higher temperature.

**Figure 13:**
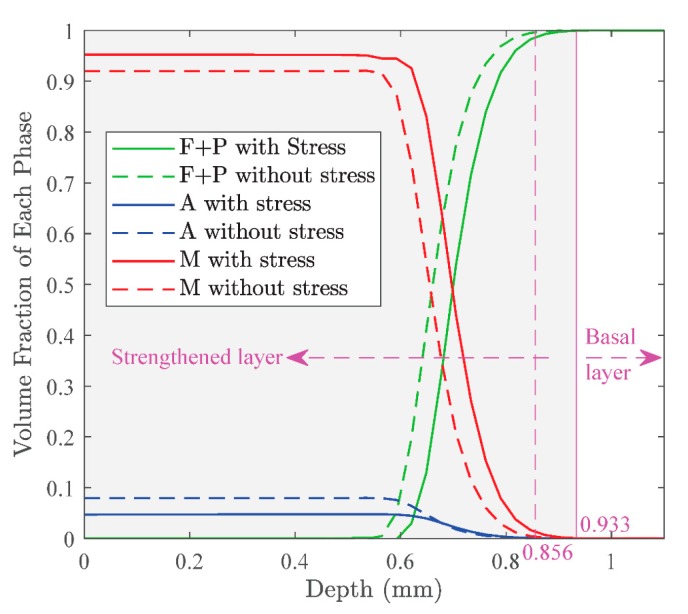
Effect of stress on the microstructure distribution and thickness of the strengthened layer.

The microstructure distribution of the strengthened layer in grinding is shown in Figure 13. As seen in the figure, the thickness of the strengthened layer was 933 μm with considering the stress, which was larger than 856 μm without considering stress. This indicated the phase transformation occurred at a deeper position with a lower temperature when considering the effect of stress. Within the strengthened layer, the fraction of martensite decreased and the fraction of ferrite + pearlite increased with the depth increasing no matter whether considering the stress or not. The figure also shows more martensite formed when considering the stress, but more austenite remained when ignoring the stress. The result illustrates that considering the effect of stress on phase transformation is necessary for predicting the microstructure distribution and thickness of the strengthened layer more accurately.

## 5. Conclusions

A thermal–mechanical–metallurgical directly coupling FE model of grinding was established. In the modeling procedure, the latent heat, volume change strain caused by phase transformation, and stress-induced phase transformation were considered. The comparison between the experimental and simulated results proved that the established model can accurately simulate the phase transformation in grinding and predict the microstructure distribution and the thickness. 

Based on the model, the evolution of the phase transformation in grinding was studied. The austenite transformation in grinding may be incomplete because the heating rate is extremely high and the duration time above A1 is short. Furthermore, the temperature drops quickly, so the undercooled austenite does not undergo other transformation before martensite transformation during the cooling period.

The effects of stress on phase transformation in grinding were revealed. Stress promotes austenite transformation because the hydrostatic stress is compressive in the period before austenite transformation and could reduce A1. The stress also promotes the martensite transformation because the hydrostatic stress is tensile in the period before martensite transformation, and both the hydrostatic tensile stress and $\sqrt{J_{2}}$ could increase Ms. 

To predict the microstructure distribution and thickness of the strengthened layer more accurately, consideration of the effects of stress on phase transformations is necessary.

## Figures and Tables

**Table 1:** Thermal properties of AISI 1045 steel.

Temp (°C)	ρ (kg/m^3^)	$C_{p}$ (J/kg °C)	k (W/m °C)
20	7850	460	49.77
100	7830	480	46.76
200	7800	498	43.24
300	7770	524	40.29
400	7740	524	37.87
500	7700	615	35.96
600	7680	690	33.18
700	7672	720	30.52
800	7660	682	27.96
900	7651	637	25.92
1000	7649	602	24.02

**Table 2:** Latent heat changes in phase transformation.

Transformation Type	$H_{x}\;(J/m^{3})$
Ferrite to austenite	595 × 10^6^
Pearlite to austenite	526 × 10^6^
Austenite to martensite	640 × 10^6^

**Table 3:** Mechanical properties of AISI 1045 steel.

Temp (°C)	E (GPa)	α (× $10{-}^{6}/{}^{\circ}C)$	*v*
20	206	11.56	0.3
100	200	11.90	
200	192	12.32	
300	184	13.09	
500	163	14.18	
800	125	14.50	
1000	99	14.40	

**Table 4:** Johnson–Cook coefficients of AISI 1045 steel.

*A*	*B*	*C*	*m*	*n*
553.1	600.8	0.0134	1	0.234

**Table 5:** Volume change ratio in phase transformation.

Transformation Type	ΔV/V
Ferrite to austenite	−3.8 × 10^−3^
Pearlite to austenite	−3.3 × 10^−3^
Austenite to martensite	1.026 × 10^−2^

**Table 6:** Chemical composition of AISI 1045 steel (wt%).

Elements	C	Si	Mn	Cr	Ni	Cu
wt%	0.42–0.45%	0.17–0.37%	0.5–0.8%	0.25%	0.3%	0.25%

**Table 7:** Experimental grinding parameters.

No.	$a_{p}$ (μm)	$v_{w}\;(m/\min)$	$v_{s}\;(m/s)$
1	450	9	30
2	400	9	
3	350	9	

## References

[1] Tang J., Du J., Chen Y. (2009). Modeling and experimental study of grinding forces in surface grinding. J. Mater. Process. Technol..

[2] Brinksmeier E., Brockhoff T. (1996). Utilization of grinding heat as a new heat treatment process. CIRP Ann. Manuf. Technol..

[3] Zarudi I., Zhang L.C. (2002). Mechanical property improvement of quenchable steel by grinding. J. Mater. Sci..

[4] Zarudi I., Zhang L.C. (2002). Modelling the structure changes in quenchable steel subjected to grinding. J. Mater. Sci..

[5] Nguyen T., Zhang L. (2010). Grinding–hardening using dry air and liquid nitrogen: Prediction and verification of temperature fields and hardened layer thickness. Int. J. Mach. Tools Manuf..

[6] Cong M., Zhou Z., Jian Z., Huang X., Gu D. (2011). An experimental investigation of affected layers formed in grinding of AISI 52100 steel. Int. J. Adv. Manuf. Technol..

[7] Liu J., Wei Y., Huang S., Xu Z. (2012). Experimental study on grinding-hardening of 1060 Steel. Energy Procedia.

[8] Yaakoubi M., Kchaou M., Dammak F. (2013). Simulation of the thermomechanical and metallurgical behavior of steels by using ABAQUS software. Comput. Mater. Sci..

[9] Wang M., Jiang S., Zhang Y. (2018). Phase transformation, twinning, and detwinning of NiTi shape-memory alloy subject to a shock wave based on molecular-dynamics simulation. Materials.

[10] Zhang L., Mahdi M. (1995). Applied mechanics in grinding-IV. The mechanism of grinding induced phase transformation. Int. J. Mach. Tools Manuf..

[11] Brosse A., Hamdi H., Bergheau J.M. (2008). A numerical study of phase transformation during grinding. Int. J. Mach. Mach. Mater..

[12] Ding Z., Li B., Liang S. (2015). Maraging steel phase transformation in high strain rate grinding. Int. J. Adv. Manuf. Technol..

[13] Deng Y., Xiu S. (2017). Research on microstructure evolution of austenitization in grinding hardening by cellular automata simulation and experiment. Int. J. Adv. Manuf. Technol..

[14] Salonitis K. (2017). A hybrid cellular automata-finite element model for the simulation of the grind-hardening process. Int. J. Adv. Manuf. Technol..

[15] Serajzadeh S. (2004). Modelling of temperature history and phase transformations during cooling of steel. J. Mater. Process. Technol..

[16] Bojinović M., Mole N., Štok B. (2015). A computer simulation study of the effects of temperature change rate on austenite kinetics in laser hardening. Surf. Coat. Technol..

[17] Guo C., Malkin S. (1994). Analytical and experimental investigation of burnout in creep-feed grinding. CIRP Ann-Manuf. Technol..

[18] Svoboda J., Gamsjäger E., Fischer F. (2005). Relaxation of the elastic strain energy of misfitting inclusions due to diffusion of vacancies. Philos. Mag. Lett..

[19] Leblond J.B., Devaux J., Devaux J. (1989). Mathematical modelling of transformation plasticity in steels I: Case of ideal-plastic phases. Int. J. Plast..

[20] Jaspers S., Dautzenberg J. (2002). Material behaviour in conditions similar to metal cutting: Flow stress in the primary shear zone. J. Mater. Process. Technol..

[21] Ding H., Shin Y.C. (2012). A metallo-thermomechanically coupled analysis of orthogonal cutting of AISI 1045 steel. J. Manuf. Sci. Eng..

[22] Avrami M. (1941). Granulation, phase change, and microstructure kinetics of phase change. III. J. Chem. Phys..

[23] Cheng H., Huang X., Wang H. (1999). Calculation of the residual stress of a 45 steel cylinder with a non-linear surface heat-transfer coefficient including phase transformation during quenching. J. Mater. Process. Technol..

[24] Fernandes F., Denis S., Simon A. (1985). Mathematical model coupling phase transformation and temperature evolution during quenching of steels. Mater. Sci. Technol..

[25] Scheil E. (1935). Anlaufzeit der austenitumwandlung. Archiv für das Eisenhüttenwesen.

[26] Koistinen D.P., Marburger R.E. (1959). A general equation prescribing the extent of the austenite-martensite transformation in pure iron-carbon alloys and plain carbon steels. Acta Metall..

[27] Griffiths B. (1987). Mechanisms of white layer generation with reference to machining and deformation processes. J. Tribol..

[28] Ramesh A., Melkote S.N. (2008). Modeling of white layer formation under thermally dominant conditions in orthogonal machining of hardened AISI 52100 steel. Int. J. Mach. Tools Manuf..

[29] Han H.N., Lee C.G., Oh C.S., Lee T.H., Kim S.J. (2004). A model for deformation behavior and mechanically induced martensitic transformation of metastable austenitic steel. Acta Mater..

[30] Denis S., Sjöström S., Simon A. (1987). Coupled temperature, stress, phase transformation calculation model numerical illustration of the internal stress evolution during cooling of a eutectoid steel cylinder. Metall. Trans. A.

[31] Denis S. (1997). Considering stress-phase transformation interactions in the calculation of heat treatment residual stresses. Mechanics of Solids with Phase Changes.

